# Tagging of Genomic STAT3 and STAT1 with Fluorescent Proteins and Insertion of a Luciferase Reporter in the Cyclin D1 Gene Provides a Modified A549 Cell Line to Screen for Selective STAT3 Inhibitors

**DOI:** 10.1371/journal.pone.0068391

**Published:** 2013-07-09

**Authors:** Andrey Samsonov, Nathan Zenser, Fan Zhang, Hongyi Zhang, John Fetter, Dmitry Malkov

**Affiliations:** Cell-Based Assays/Reporter Cell Lines, Sigma-Aldrich, St. Louis, Missouri, United States of America; Center for Genomic Regulation, Spain

## Abstract

Signal transducer and activator of transcription 3 (STAT3) is an oncogenic protein that is constitutively activated in numerous cancer cell lines and human cancers. Another STAT family member, STAT1, possesses cancer-inhibitory properties and can promote apoptosis in tumor cells upon activation. To better characterize these important cancer related genes, we tagged STAT3 and STAT1 loci with fluorescent protein (FP) sequences (RFP and GFP respectively) by targeted integration via zinc finger nuclease (ZFN) - mediated homologous recombination in A549 cells that express aberrantly activated STAT3. We inserted the FP transgenes at the N-terminus of the STAT3 locus and at the C-terminus of the STAT1 locus. The integration resulted in endogenous expression of fluorescent STAT3 and STAT1 chimeric fusion proteins. When stimulated with IL-6 or IFN-γ, the cells showed robust nuclear translocation of RFP-STAT3 or STAT1-GFP, respectively. Pre-incubation of cells with a known specific STAT3 inhibitor showed that IFN-γ-induced translocation of STAT1-GFP was not impaired. STAT3 activates multiple downstream targets such as genes involved in cell cycle progression - e.g. cyclin D1. To detect changes in expression of endogenous cyclin D1, we used ZFN technology to insert a secreted luciferase reporter behind the cyclin D1 promoter and separated the luciferase and cyclin D1 coding regions by a 2A sequence to induce a translational skip. The luciferase insertion was made in the RFP-STAT3/STAT1-GFP cell line to have all three reporters in a single cell line. Addition of a STAT3 inhibitor led to suppression of cyclin D1 promoter activity and cell growth arrest. The triple-modified cell line provides a simple and convenient method for high-content screening and pre-clinical testing of potential STAT3 inhibitors in live cells while ensuring that the STAT1 pathway is not affected. This approach of reporting endogenous gene activities using ZFN technology could be applied to other cancer targets.

## Introduction

Human genome manipulation has become a powerful tool for understanding the mechanisms of numerous diseases including cancer. This approach is also very promising for anti-cancer drug screening when a model cell line with specific modified genes is used to robustly and effectively discover novel small molecule drugs. The modifications can allow monitoring endogenous gene activities by inserting reporter sequences in the desired locations in the genome.

Tagged proteins are used extensively to provide a visual readout in cells. Uses of tagged proteins include the study of protein abundance and localization, transcriptional and translational regulation, posttranslational modifications, protein–protein interactions, alternative splicing, RNAi-dependent effects, and others. However, current methods of expressing tagged proteins in the cell can result in distorted expression that does not accurately reflect the expression pattern of the endogenous locus. Expression of tagged proteins often relies on heterologous promoters for expression. In addition, some tagged proteins are expressed from episomal or randomly integrated vectors and are therefore not controlled by the endogenous regulatory pathways leading to nonphysiological expression patterns. Thus there exists a strong need for a method that can direct specific integration into a chromosome of a cell to produce a tagged protein controlled by endogenous regulatory pathways. One way to achieve this targeted integration into the genome is by using zinc finger nucleases (ZFNs).

Classical ZFNs are fusions of zinc finger proteins (ZFPs) and the catalytic DNA-cleavage domain of FokI, a type II endonuclease. The zinc finger domain confers binding affinity and specificity while the nuclease domain dimerizes and cleaves the DNA to generate a double-strand break (DSB). The cell then employs the natural DNA repair mechanisms of either error-prone non-homologous end-joining (NHEJ), single-strand annealing (SSA), or high-fidelity homologous recombination (HR) [1]. Consequently, ZFNs facilitate efficient targeted editing of the genome by creating DSBs at user-specified locations. ZFNs have been primarily used to create gene knockouts in mammalian cell lines and various species including zebrafish, rats, flies, and worms utilizing NHEJ or SSA [2]. Here we relied on the HR repair pathway which has been used for tagging of various genes in different cell lines [3–5]. We have used ZFN-mediated reporter insertion to probe native STAT signaling in live cells.

STATs are transcription factors that mediate signaling by cytokines [6,7]. Following type I IFN (IFN-α and IFN-β) or type II IFN (IFN-γ) binding to cell surface receptors, Jak kinases (TYK2 and JAK1) are activated, leading to tyrosine phosphorylation of STATs. The phosphorylated STATs dimerize, associate with ISGF3G/IRF-9 to form a complex termed ISGF3 transcription factor that translocates into the nucleus. ISGF3 binds to the IFN-stimulated response element (ISRE) to activate the transcription of multiple interferon stimulated genes that drive the cell in an antiviral and/or anti-cancer state [8,9]. There are seven identified STATs: STAT1, STAT2, STAT3, STAT4, STAT5A, STAT5B, and STAT6 [10].

STAT3 is constitutively activated in numerous cancer cell lines and in many solid and hematological human cancers, including multiple myeloma, several lymphomas and leukemias, breast cancer, prostate cancer, ovarian carcinoma, melanoma, renal carcinoma, and colorectal carcinoma [11,12]. This activation appears to be caused by changes upstream in the pathway leading to elevations in STAT3 phosphorylation [11]. Mutations in STAT3 leading to constitutive activation are not found in naturally occurring tumors [11]. To study the effect of just increasing STAT3 activity, site-directed mutagenesis was used to replace two residues with cysteines within the C-terminal loop of the SH2 domain of STAT3. This substitution produces a molecule (termed STAT3-C) that dimerizes spontaneously [13]. The expression of STAT3-C in immortalized fibroblasts causes cellular transformation scored by colony formation in soft agar and tumor formation in nude mice [13]. Because of this oncogenic potential, STAT3 has been studied as a therapeutic target for cancer [14]. The inhibition of STAT3 induces apoptosis in melanoma cells and suppresses the growth of head and neck tumors in nude mice [15,16].

On the contrary another STAT family member - STAT1 is anti-oncogenic and it promotes apoptosis in tumors by inducing the expression of cell surface death receptors and their ligands. In response to IFN-γ stimulation, STAT1 forms homodimers that bind to the **GAS** (Interferon-Gamma Activated **S**equence) promoter element. In response to either IFN-α or IFN-β stimulation, STAT1 forms a heterodimer with STAT2 that can bind the ISRE (Interferon Stimulated Response Element) promoter element [17]. In either case, binding of the promoter element leads to an increased expression of ISG (Interferon Stimulated Genes). STAT1 is also known to act as a negative regulator of tumor growth and metastasis by playing a key role in the inhibition of angiogenesis [18]. Therefore, a potential anti-cancer drug candidate should selectively inhibit STAT3 function as a transcription activator while not affecting STAT1 (and other STAT family members).

According to a recently published review there are two main strategies to inhibit the STAT3 signaling pathway: 1) indirectly, i.e. by blocking the upstream molecules, or 2) directly by targeting STAT3 protein [12,19]. The first approach includes blocking the cytokine/receptor binding and/or the inhibition of Jak kinases. Such approaches may induce several undesirable events as Jak kinases phosphorylate multiple substrates in the cell. A better approach might be to directly target STAT3 and prevent such events as protein phosphorylation, dimerization, nuclear translocation or STAT3-ISRE binding. Finding a selective inhibitor of the binding between the STAT3 dimer and the ISRE sequence is challenging due to the interference with STAT1 and STAT2 as their heterodimers also bind the ISRE after stimulation with Type I interferons [17]. It would be easier to prevent any of the first three steps of STAT3 activation (phosphorylation, dimerization and nuclear translocation mediated by nuclear shuttling transport factors importins [20]) that lead to the abrogation of the nuclear translocation event. The translocation leads to massive re-distribution of STAT3 from the cytoplasm into the nucleus shortly after stimulation (15-30 minutes). If tagged with fluorescent proteins, STAT redistribution is easy to observe and quantify with high-content screening and analysis software [21]. The nuclear translocation readout could provide tremendous leverage in identifying highly selective STAT3 inhibitors. Any chemical compounds that effectively stop STAT3 nuclear translocation in live cells should be qualified as strong leads. A next step might be to further elucidate the underlying mechanisms (e.g. phosphorylation, dimerization or importin inhibition).

STAT3’s SH2 domain participates in binding to the phosphorylated gp130 subunit of the cytokine receptor complex as well as in STAT3 dimerization [6]. So a small molecule that can penetrate the cell membrane and directly and selectively bind the STAT3 SH2 domain should block STAT3 phosphorylation and pSTAT3:pSTAT3 dimerization [22]. As SH2 domains in different STATs exhibit high homology, the STAT3 SH2 domain based inhibitors should have much higher affinity for STAT3 SH2 to be specific. Then other STATs activation (such as STAT1) should not be affected.

Activated STAT3 binds to DNA and activates transcription of multiple downstream genes linked to cell cycle progression (Cyclin D1, Fos, c-Myc, etc.) and inhibition of apoptosis (Survivin, Bcl-2 and Bcl-xL) [12,23,24]. Being able to track one of these downstream targets along with effects on STAT3 and STAT1 should allow for better characterization of compounds during screening. This led to our design of a model system allowing for observation of endogenous STAT3/STAT1 nuclear translocation and reporting of Cyclin D1 (CCND1) promoter activity simultaneously in live cells in real time. This system would allow searching for selective STAT3 inhibitors without perturbing the native gene regulation. A compound’s delivery and toxicity can be controlled at the same time as well.

## Materials and Methods

### Chemicals

Unless otherwise indicated, all reagents and materials used in this work were obtained from Sigma-Aldrich (St. Louis, MO): IFN-γ (I3265), IL-6 (206-IL-010, R&D Systems, Inc), IL-10 (H7541), EGF (E9644), IFN-β-1a (I4151), TGF-α (T7924), TGF-β1 (T1654), Stattic (S7947), Hoechst 33342 (B2261). Cpd3 (4-[3-(2,3-dihydro-1,4-benzodioxin-6-yl)-3-oxo-1-propen-1-yl] benzoic acid) was ordered through MolPort (MolPort ID: MolPort-001-889-847, supplier ChemDiv, catalog # 4031-0592).

GFP and TagGFP2, and RFP and TagRFP are synonymous for the fluorescent reporter genes in this manuscript. The GFP and RFP are monomeric and were obtained from Evrogen, referred to as TagGFP2 and TagRFP respectively [25].

### Parental cell line and culture conditions

Human lung carcinoma cell line A549 (CCL-185) was obtained from American Type Culture Collection (ATCC) and grown in RPMI-1640 medium (R0883) supplemented with 2 mM L-glutamine (G7513) and 10% fetal bovine serum (F2442) in 5% CO_2_ in air atmosphere, 37°C. Trypsin-EDTA solution (T3924) was used for sub-culturing.

### ZFNs and donors - design and production

CompoZr® ZFNs were designed and manufactured by Sigma-Aldrich applying bioinformatic tools to assure that the ZFN binding site is unique (4 or more mismatches for a 24 base pair recognition site) within the genome [26]. High ZFN specificity was also guaranteed by using obligate heterodimer FokI cleavage domains [27]. The CEL-I enzymatic mutation detection assay (706020, Transgenomic, Inc) was used to assess ZFN cutting efficiency [28].

**Figure 1:**
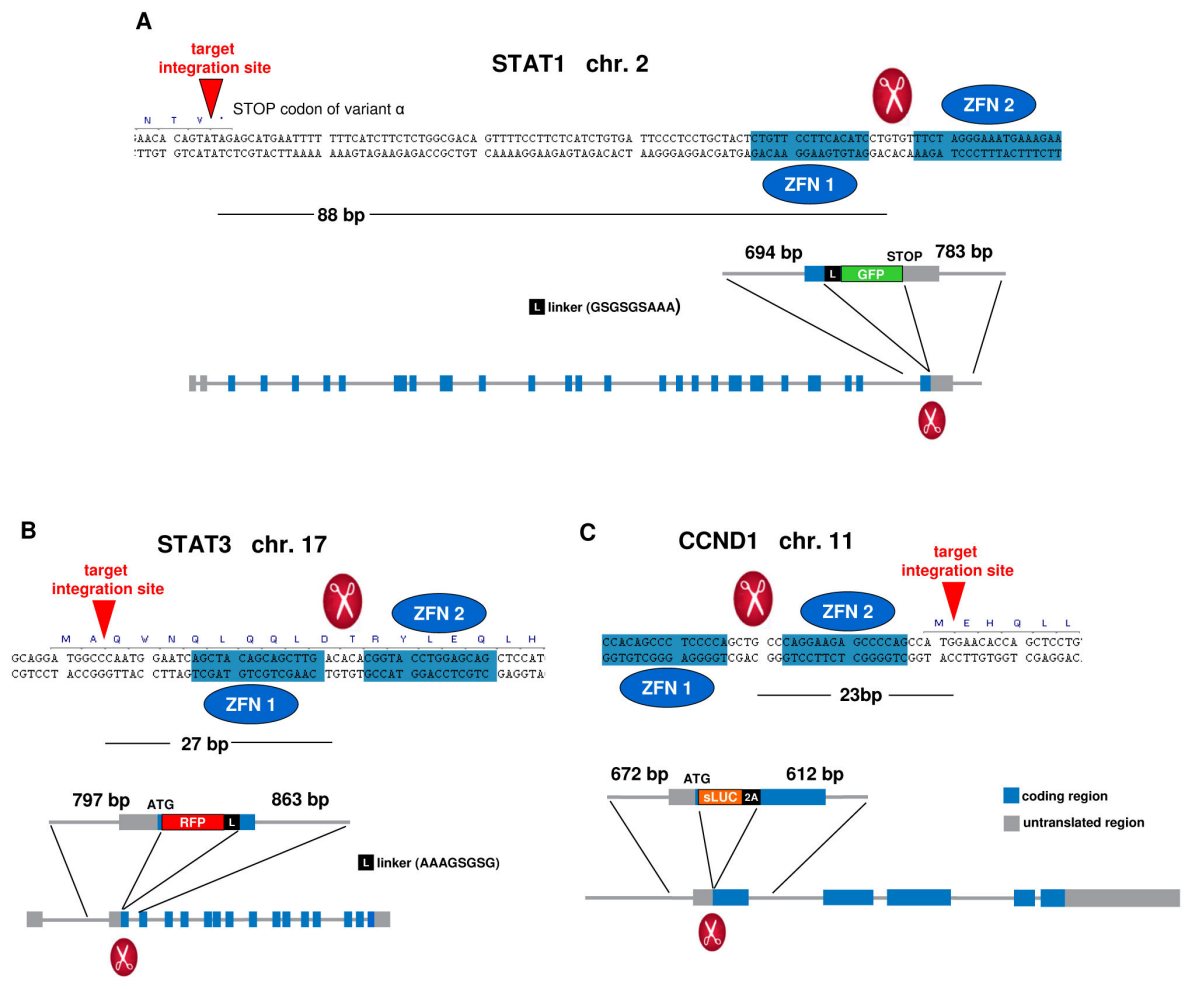
Schematics of the STAT1 (A), STAT3 (B) and CCND1 (C) tagged loci. The gene sequence and relative positions for the ZFN binding site, ZFN cut site, and the targeted integration site are shown. The integration position of the donor relative to the introns and exons of the gene is indicated in the lower schematic. The donor design is shown with the homology arms of indicated length and the corresponding insert.

Up to 100 bp was allowed between the cut and integration sites (Figure 1). It is known that genome editing can be done ~100 bp away from the cut site without drastic drops in efficiency [29,30].

Donors were synthesized by GenScript with ~700 bp of each homology arm. A short linker consisting of small hydrophilic amino acids (a few copies of alternating glycine and serine [31]) was placed between the FP and the target protein to improve its accessibility. For insertion of a secreted luciferase into the CCND1 locus, the linker was replaced by a translational skip sequence (2A) [32,33] (Figure 1).

The location of the FP-insert (N vs C terminus of the gene of interest) was chosen to ensure that the resulting FP-fusion does not block the normal localization and functionality of the protein of interest. This requirement placed some constrains on whether all splice variants / isoforms of the target gene are tagged: in the case of STAT1 the C-terminal insertion of GFP tags only its α isoform while all STAT3 isoforms are tagged by inserting the RFP N-terminally (Figure 1).

### Nucleofection and clone isolation

Donor constructs (1 µg per kb) containing each insert flanked by sequences homologous to the regions on either side of the genomic target site were nucleofected along with ZFN mRNA (1 µg for each ZFN in the pair) designed to cut near the genomic target site (Figure 1) into 1 million A549 cells with the Amaxa® Nucleofector® device (Cat. No. AAD-1001) and Nucleofector® Kit T (Cat. No. VCA 1002) from Lonza AG according to the product manual. The mRNA was prepared using the following: C-MMA60710, MessageMAX™ T7 ARCA-Capped Message Transcription Kit, CELLSCRIPT™, Inc.; AM1908, Ambion® MEGAclear™ Kit, Life Technologies; PAP5104H, Poly(A) Polymerase Tailing Kit, Epicentre Biotechnologies. The cold shock procedure [34] was used for ZFNs with low activity to boost their cutting efficiency.

**Figure 2:**
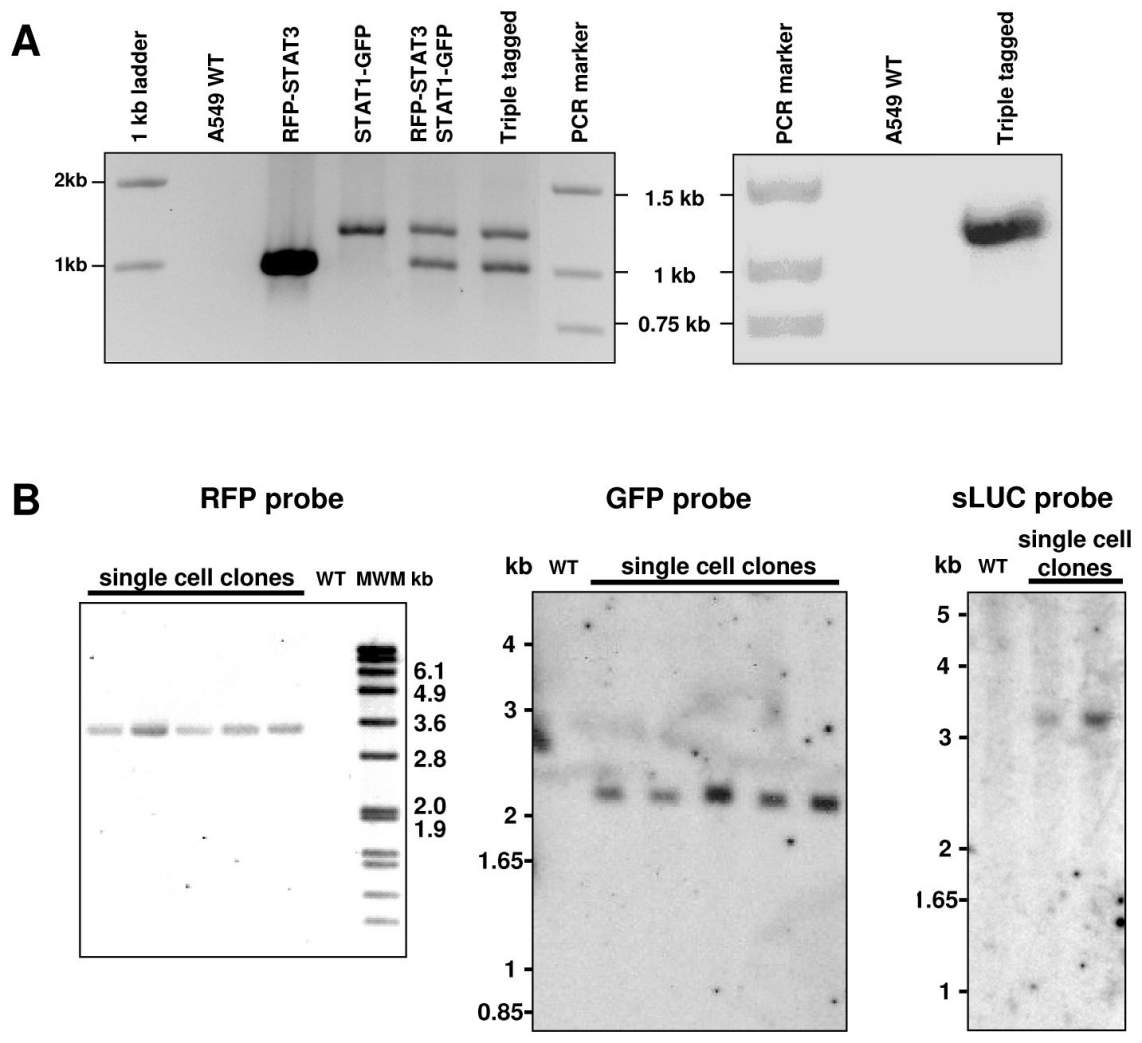
Tests for correct reporter integration. A. Junction PCR analysis checks for correct donor integration. PCR was performed using one primer specific to the insert sequence and the other specific to the genomic sequences outside of the homology arms. Left panel: Multiplex junction PCR performed with two pairs of primers to detect the integration of RFP and GFP simultaneously. Genomic DNA samples isolated from the parental cells (A549WT), single-tagged single cell clones (RFP-STAT3 and STAT1-GFP), a double-tagged clone (RFP-STAT3/STAT1-GFP) and the triple-tagged clone (RFP-STAT3/STAT1-GFP/sLUC-2A-CCND1) were analyzed. Expected amplified fragments: 926bp (RFP-STAT3) and 1144bp (STAT1-GFP). Right panel: Junction PCR to detect the luciferase integration in the Cyclin D1 locus (expected fragment is 1252bp) in the triple-tagged clone (RFP-STAT3/STAT1-GFP/sLUC-2A-CCND1). No PCR product was detected in the wild type control. Junction PCR products were confirmed by sequencing.

The triple integration was done in three rounds. During the first round the cells were nucleofected with the STAT3 ZFNs and a corresponding RFP-containing donor. Integration resulted in endogenous expression of fluorescent fusion protein RFP-STAT3. The cells were RFP-sorted to single cells by flow cytometry (BD Biosciences FACS Aria III) and expanded into clonal populations. Testing of the clones was performed to select a single RFP-STAT3 clone as a stable cell line (Figure 2A). This line was used as starting material for the second round of integration in which nucleofection was carried out with the STAT1 ZFNs and a corresponding GFP-containing donor. A single GFP-STAT1 clone was isolated similar to the first round, resulting in generation of the double knockin reporter line GFP-STAT1/RFP-STAT3 (Figure 2A). In the third round the luciferase insertion into the Cyclin D1 locus was done in RFP-STAT3/STAT1-GFP cells and triple knock-in RFP-STAT3/STAT1-GFP/sLUC-2A-CCND1 clones were isolated based on luminescence.

B. Southern blotting tests for possible random integration of the plasmid donors used for genome editing. Genomic DNA from single cell clones and from A549 wild type (WT - served as a negative control) were digested with restriction endonucleases *Xba*I / *Sph*I (for detection of RFP off-target insertion), *Nde*I (for detection of GFP off target insertion) or *EcoR* I (for detection of sLUC off-target insertion). DIG-labeled RFP probe or Radioactive-labeled GFP and sLUC probes were used. Proper targeted insertion of the RFP into the STAT3 locus should produce a hybridized band of approximately 3.3 kb in size, GFP into the STAT1 locus - approximately 2.1 kb in size, sLUC-2A into the CCND1 locus - approximately 3.0 kb in size.

Junction PCR showed at least one allele was tagged for each target (Figure 2A) and Southern hybridization analysis showed there were no off-target insertions of the reporter sequences (Figure 2B). The correct donor integration was ensured by sequencing at least one of the junctions for each insertion (data not shown).

### Junction PCR analysis

Junction PCR was performed on a Veriti 96-well Thermal Cycler (Applied Biosystems). For multiplex PCR to detect integrations of RFP into the STAT3 locus and GFP into the STAT1 locus, JumpStartTM TEDTaqTM ReadyMixTM PCR Reaction Mix (P0982) was used for PCR amplification. For Luciferase integration in the CCDN1 locus, junction PCR was performed using AccuPrimeTM Taq DNA Polymerase System (1083000, Invitrogen).

Junction PCR primer set sequences:

RFP-STAT3 (right junction)

forward RFP aagagacctacgtcgagcagc reverse STAT3 aagaagaaagaaggctggatgtagc

STAT1-GFP (left junction)

forward STAT1 tggtgctactggtattcggggct reverse GFP gtcttgtacttgccgtcgtcct

sLUC-2A-CCND1 (right junction)

forward sLUC ctggaggtgctgatcgagatggag reverse CCND1 ggcgcataatactggcacgagc

### Imaging and image analysis

Fluorescent imaging of cells was done with a Nikon Eclipse TE2000-E inverted research microscope, a Photometrics CoolSNAP ES^2^ cooled CCD camera, and MetaMorph® software using a 40x/1.3 oil objective. Cells were imaged live in Hanks’ balanced salt solution (H8264) supplemented with 2% fetal bovine serum (F2442). Filtersets were GFP (exs 450–490/em 500–550) and RFP (exs 530–560/em 590–650). The redistribution of STAT1-GFP and RFP-STAT3 fusion proteins over time was quantified using translocation journals in MetaMorph®. Less than 1 µM Hoechst 33342 was used as a nuclear marker if necessary (exs 395-410/em 430-480).

### Western Blot Analysis

Cells were lysed using CelLytic M (C2978) containing proteinase (S8820) and phosphatase (P5726 and 04906845001, Roche) inhibitors. Total Protein concentrations were determined using a bicinchoninic acid assay (BCA1). Samples (50 to 100 µg) were separated electrophoretically under reducing conditions in a 6% Tris-glycine gel and transferred to a nitrocellulose membrane. After transfer, membranes were blocked with 5% BSA (A9647) in TBST (T9039) overnight and probed with primary antibodies: GFP (AB121; Evrogen), RFP (AB234; Evrogen), phosphorylated STAT1 Y701 (clone D4A7; Cell Signaling), phosphorylated STAT3 Y705 (clone 9E12; Santa Cruz), total STAT3 (9132S; Cell Signaling), and α-tubulin (T5168) for two hours. Subsequently, the membrane was incubated with the corresponding secondary-conjugated horseradish peroxidase (anti-mouse (A4416) or anti-rabbit (A0545)) for one hour before the addition of the chemiluminescent peroxidase substrate (CP1300). Imaging was done with a Chemidoc XRS System (Bio-Rad).

### Southern Blot Analysis

Southern Blot Hybridization was performed using DIG-labeled RFP probe for RFP-STAT3, or ^32^P-labeled GFP-probe and luciferase probe for STAT1-GFP and sLUC-CCND1, respectively, in single isolated clones. Total genomic DNA was isolated using GenElute Mammalian Genomic kit (G1N70-1kt). A total of 5-10 µg of restriction enzyme digested genomic DNA was used for each sample for Southern Blot analysis. DIG-labeled probe was produced using Roche’s PCR DIG Probe Synthesis Kit (product 11 636 090 910) and probe hybridization and detection was performed by Roche’s DIG Wash & Block Buffer Set (product 11 585 762 001). Imaging was done with a Chemidoc XRS System (Bio-Rad). For ^32^P-based Southern Blot analysis, restriction enzyme digestion DNA was shipped to Lofstrand Labs Limited (Gaithersburg, MD) to complete the analysis.

### Luminescence measurements

We used a secreted luciferase, sLS7C25, licensed from GeneStream [35]. It is a synthetic luciferase with homology to the naturally occurring *Gaussia* and *Metridia* luciferases. All three use coelenterazine substrate. Aliquots of the supernatant were analyzed on a SpectraMax L luminometer (Analytical Technologies) with the RapidReporter® *Gaussia* Luciferase assay (ActiveMotif, Cat. No. 33001).

## Results

## Summary

**Figure 3:**
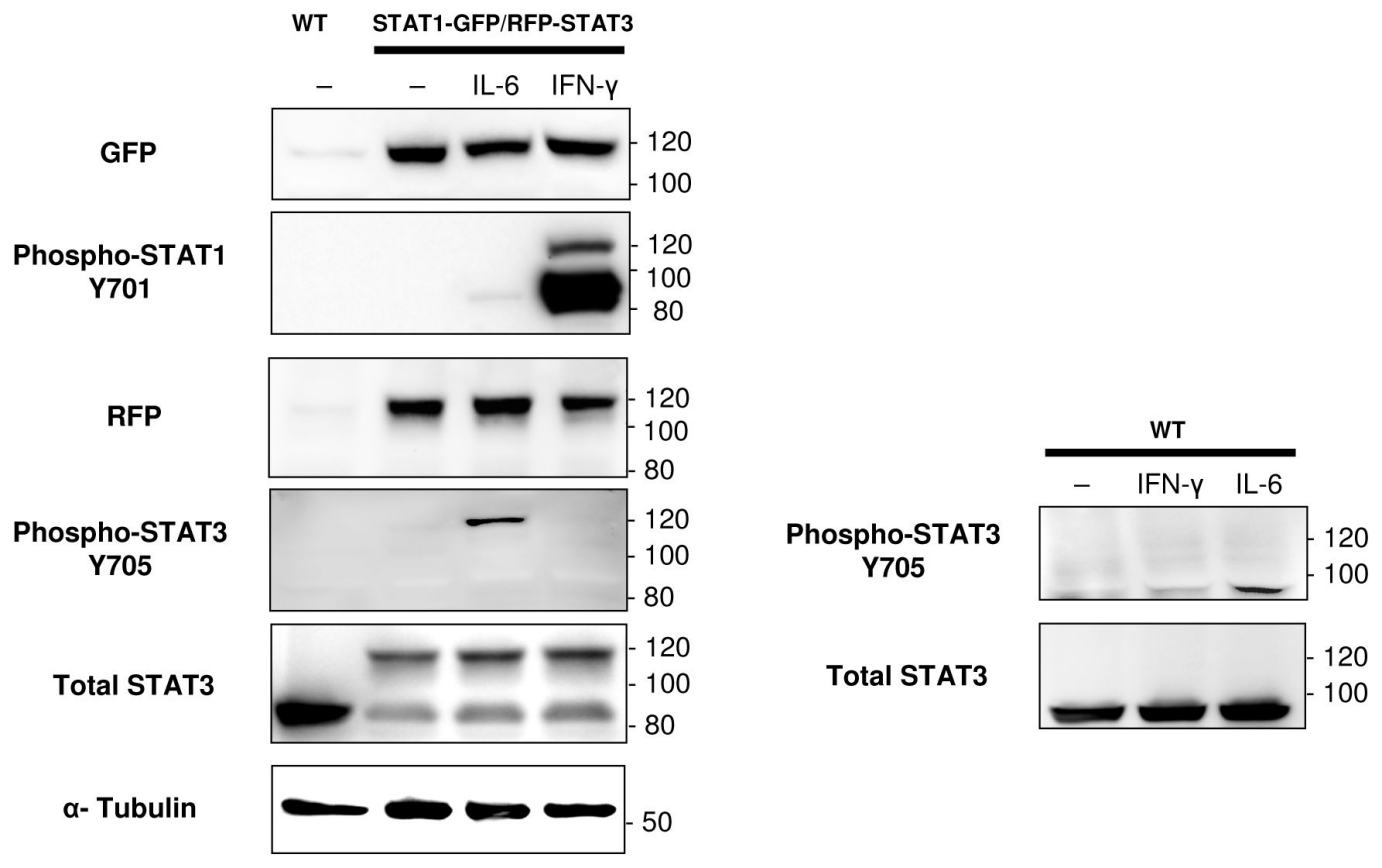
The targeted FP integration leads to endogenous expression of the corresponding fusion proteins. Wild type (WT) and ZFN modified (STAT1-GFP/RFP-STAT3) cell lysates were analyzed by Western blot with and without STAT1/STAT3 activation using antibodies recognizing epitopes on FP tags and phosphorylated and total STATs. α-Tubulin or total STAT3 levels were analyzed as loading controls. The molecular weights of unmodified and FP-tagged STATs are around 90 and 120 kDa, respectively. At least one copy was tagged and at least one copy was untagged for each STAT. In the case of STAT1, expression of only its major isoform STAT1α lead to STAT1-GFP fusion protein production while all STAT3 isoforms were tagged if expressed off the gene copy with the FP insert.

**Figure 4:**
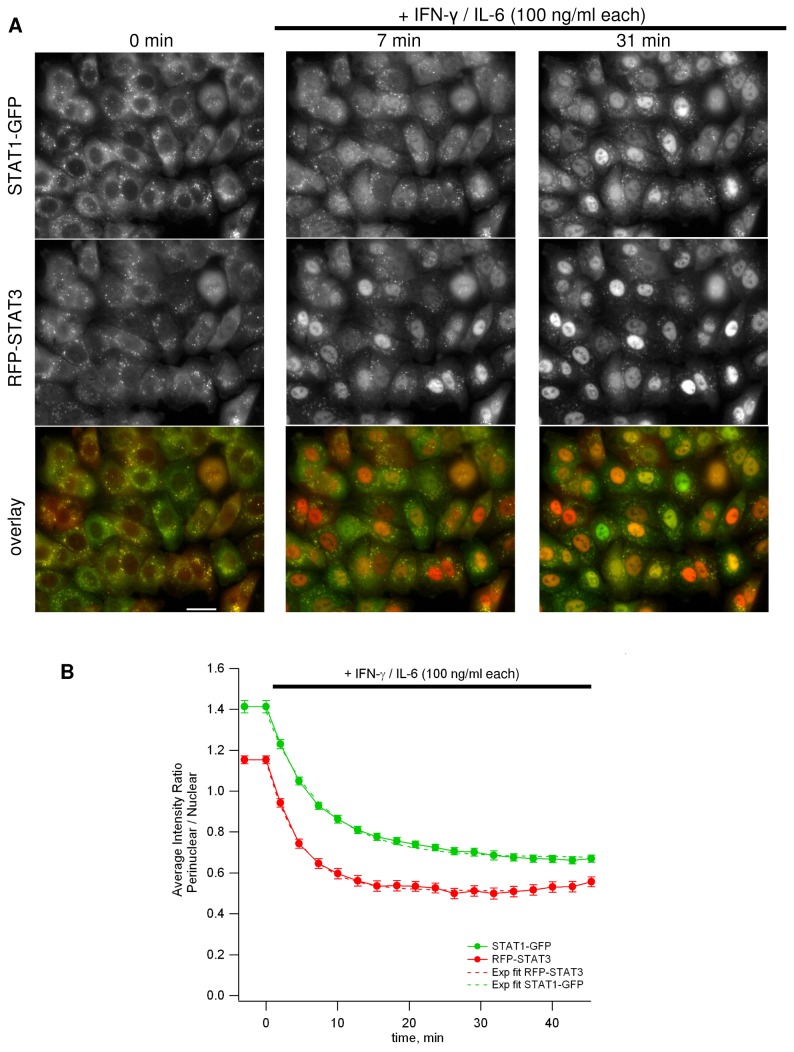
Endogenous STAT1/STAT3 nuclear translocation upon simultaneous activation. **A**. Fluorescence microscopy images of an isolated single cell clone expressing the endogenous STAT1 protein tagged with GFP at the C-terminus and the endogenous STAT3 protein tagged with RFP at the N-terminus (A549 lung carcinoma). The cells were imaged live before and after addition of 100 ng/mL IFN-γ and 100 ng/mL IL-6 using a 40x/1.3 oil objective. The scale bar is equal to 25 µm. **B**. The nuclear redistribution of both fusion proteins over time was quantified using translocation analysis in MetaMorph. Mean values ± SE, n = 50 are shown. Exponential fits gave the following decay time (±SD): τ_STAT1_ = 7.6 ± 3.0 min, τ_STAT3_ = 4.8 ± 2.5 min.

**Figure 5:**
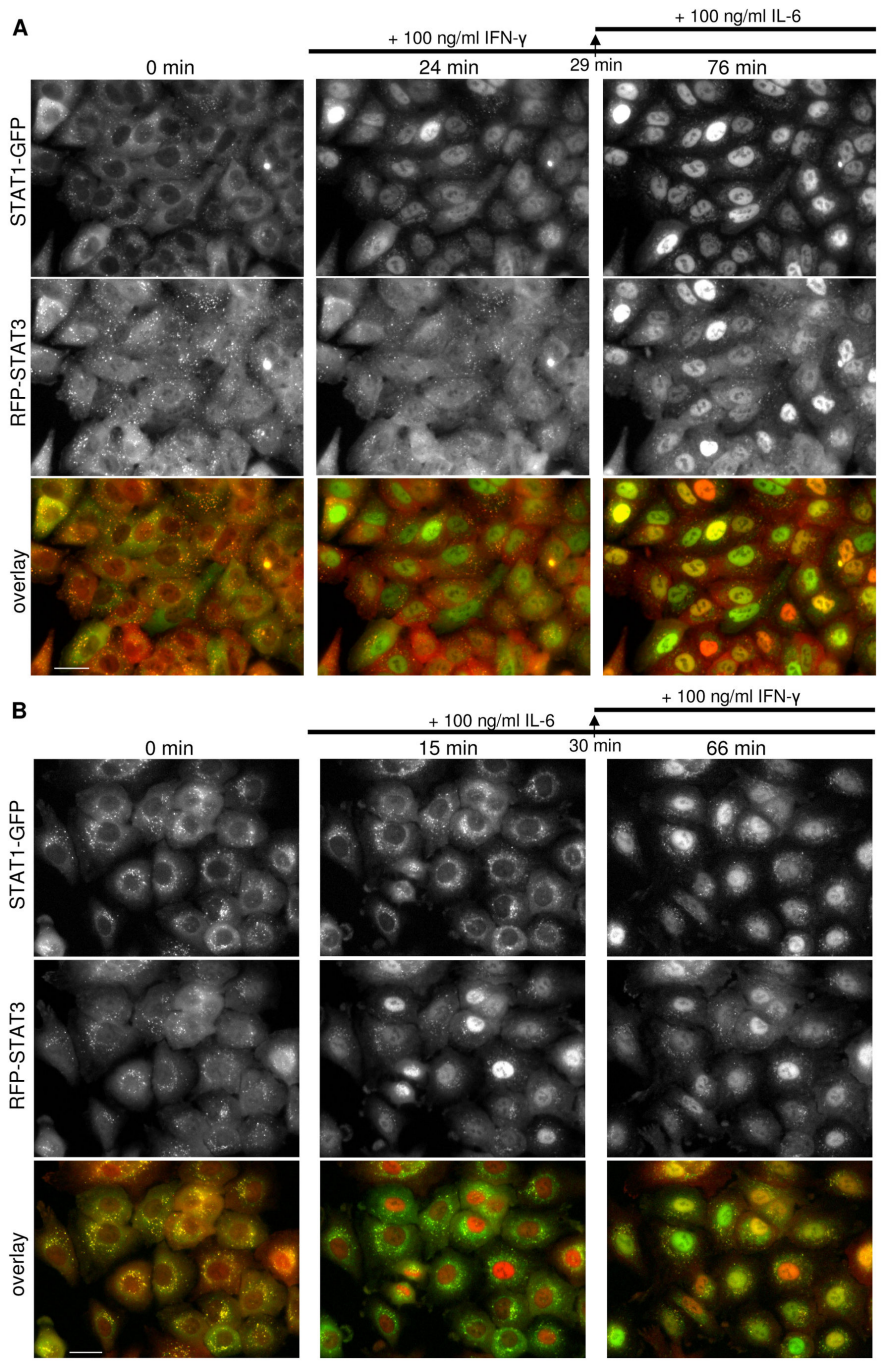
Upstream ligand selectivity for activation of endogenous STAT1/STAT3. Fluorescence microscopy images of an isolated single cell clone expressing the STAT1 gene endogenously tagged with GFP at the C-terminus and the STAT3 gene endogenously tagged with RFP at the N-terminus (A549 lung carcinoma). The cells were imaged live a using 40x/1.3 oil objective. The scale bar is equal to 25 µm. **A**. The STAT1-GFP and RFP-STAT3 images were taken before and 24 minutes after addition of 100 ng/mL of IFN-γ. 100 ng/mL of IL-6 was added 29 minutes after IFN-γ and final images were taken 47 minutes after IL-6 addition. **B**. The STAT1-GFP and RFP-STAT3 images were taken before and 15 minutes after addition of 100 ng/mL of IL-6. 100 ng/milliliter of IFN-γ was added 30 minutes after IL-6 and final images were taken 36 minutes after IFN-γ addition.

**Figure 6:**
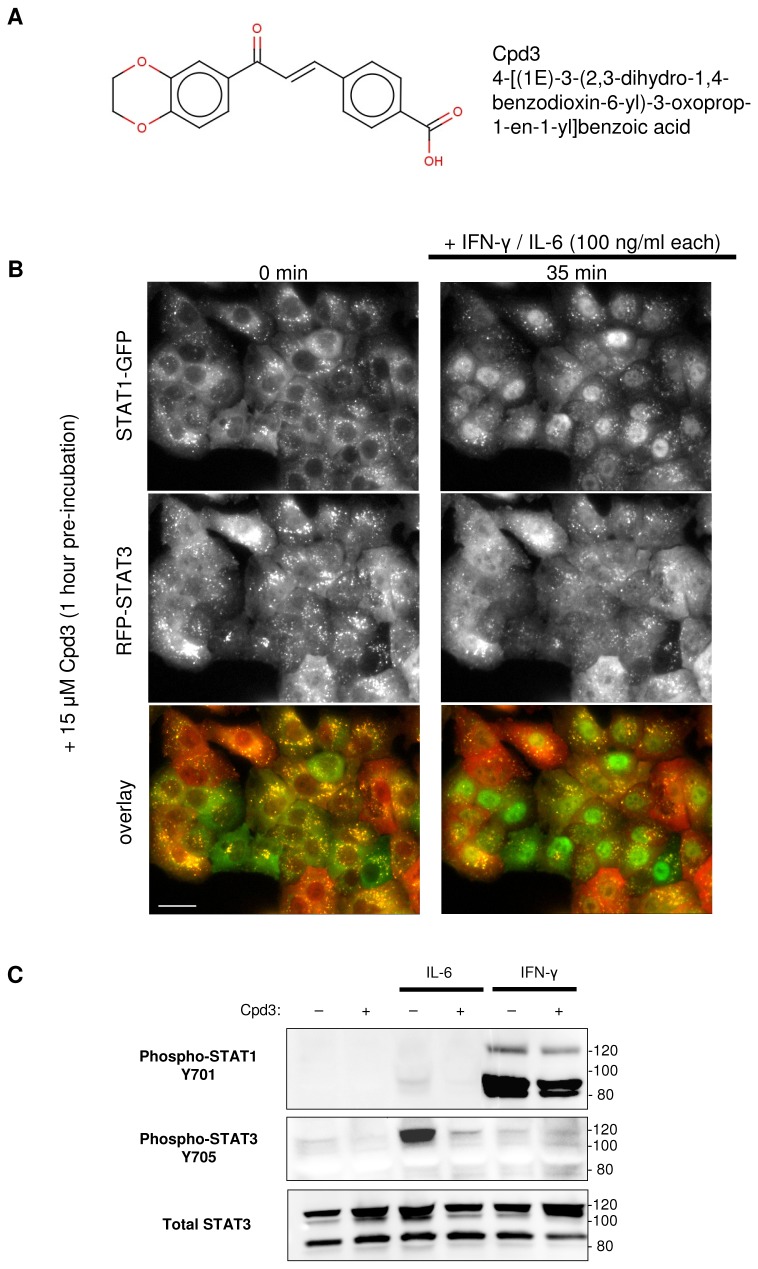
Cpd3 selectively inhibits activation of endogenous STAT3, not STAT1. **A**. Cpd3 structure. **B**. Fluorescence microscopy images of an isolated single cell clone expressing the endogenous STAT1 protein tagged with GFP at the C-terminus and the endogenous STAT3 protein tagged with RFP at the N-terminus (A549 lung carcinoma). Cells were pre-incubated for 1 hour with 15 µM Cpd3, a specific STAT3 inhibitor. The addition of a mixture of 100 ng/ml each of IL-6 and IFN-γ did not induce STAT3 nuclear translocation, but STAT1 translocated into the nucleus in the usual manner. The STAT3 and STAT1 images were taken 35 minutes after addition of the receptor ligands. The cells were imaged live using a 40x/1.3 oil objective. The scale bar is equal to 25 µm. **C**. ZFN modified (STAT1-GFP/RFP-STAT3) cell lysates were analyzed by Western blot using antibodies recognizing epitopes on phosphorylated STATs and total STAT3. Before lysis cells were or were not pre-incubated for 1 hour with 15 µM Cpd3, with and without consecutive STAT3/STAT1 activation (100 ng/ml IL-6 / IFN-γ for 30 minutes). Total STAT3 levels were analyzed as loading controls. The molecular weights of unmodified and FP-tagged STATs are around 90 and 120 kDa respectively.

**Figure 7:**
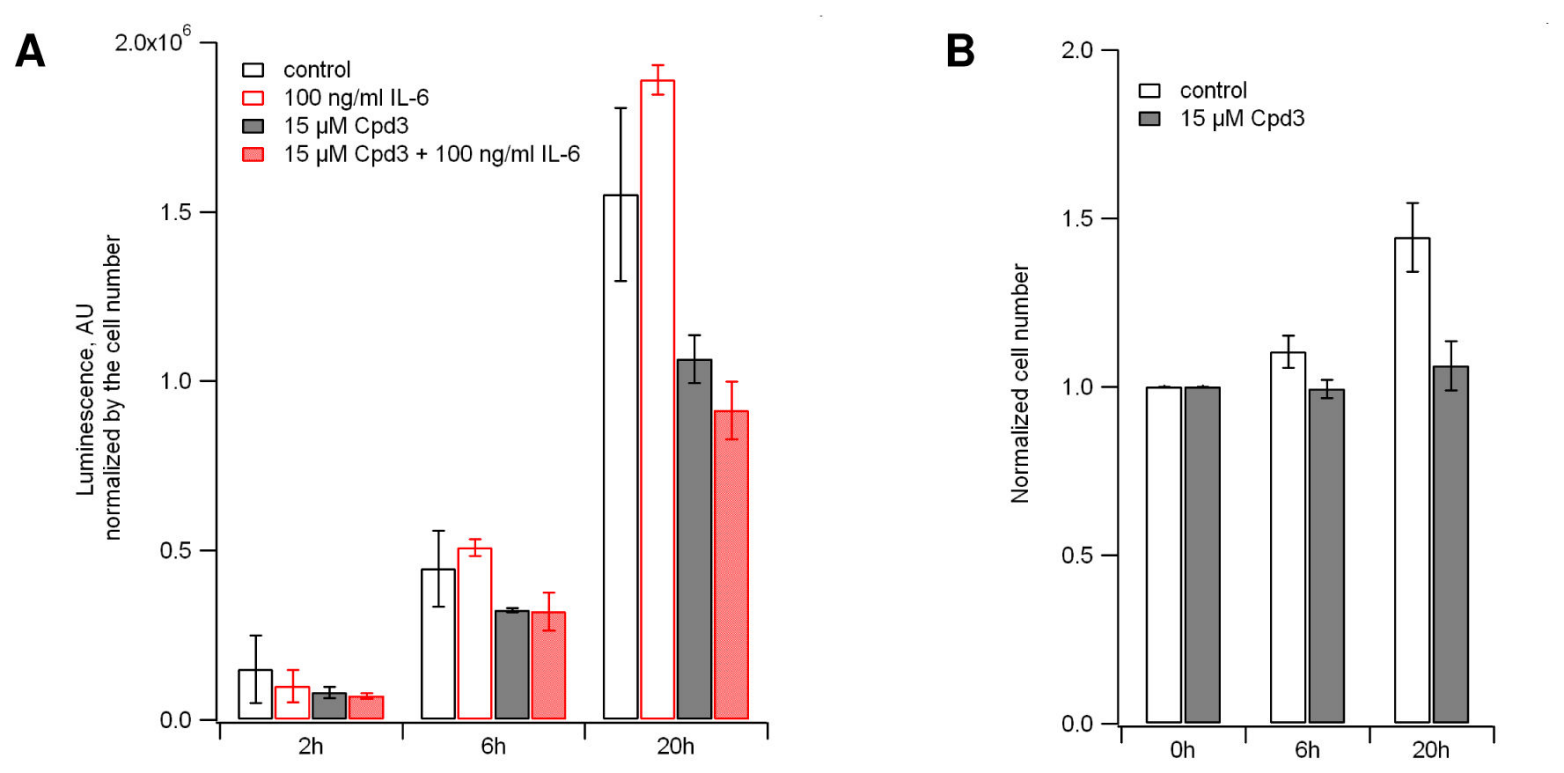
Cpd3 down-regulates Cyclin D1. **A**. Cpd3 effect on Cyclin D1 expression determined by luminescence signal in A549 RFP-STAT3/STAT1-GFP/sLUC-2A-CCND1 cells with and without IL-6 stimulation. The experiment was started after changing the media and adding IL-6 if required. Cpd3 treated cells were pre-incubated with Cpd3 for 1 hour before that. The media was analyzed at the indicated time points and the readings were normalized by the cell number. The mean ± SD is plotted, n = 3. **B**. Cpd3 blocks A549 cell division (see also Movie S5). Control and Cpd3 treated cell counts were normalized at 0 hours. Cpd3 treated cells were pre-incubated with Cpd3 for 1 hour before starting the experiment. The mean ± SD is plotted, n = 6.

We used ZFN technology to integrate fluorescent reporter sequences (RFP and GFP) into the endogenous loci of STAT3 and STAT1 in A549 cells. We also inserted a secreted luciferase into the CCND1 (Cyclin D1) locus to place the reporter under the Cyclin D1 promoter with a translational skip spacer (2A) [32,33] between the luciferase and CCND1 coding region. All three gene loci were modified in the same cell line to allow simultaneous real-time reporting of their endogenous activity (Figure 1). Single cell knock-in clones were isolated containing one correctly targeted allele, with no off-target donor insertion (Figure 2). The FP integrations resulted in expression of the corresponding genes’ fusion proteins (Figure 3 Movie S1). Their cytokine-induced nuclear translocation could be monitored (Figures 4-5 Movie S2-S3) and quantified (Figure 4B) using the fraction localized in the nucleus (FLIN) measurement [21,36]. We used Cpd3 [36] as an example compound to test STAT3 inhibitory activity in the generated reporter knock-in line by monitoring RFP/GFP fluorescence (Figure 6 Movie S4) and by analyzing luciferase signal in the culture medium (Figure 7A). We also monitored cell growth for a day (DIC time-lapse every 5 minutes) in the presence of Cpd3 in the media to observe the compound’s effect on the cell reproduction cycle (Figure 7B Movie S5).

### Testing for the endogenous activity of STAT3 and STAT1

CompoZr® ZFN technology allowed us to develop the knock-in reporter cell line expressing the endogenous STAT3 protein tagged with RFP at the N-terminus and the endogenous STAT1 protein tagged with GFP at the C-terminus (Figure 1). Western (Figure 3) and fluorescence imaging analysis (Figures 4-5) of these fusion proteins indicated that both STAT3 and STAT1 native gene regulation is conserved resulting in normal levels of protein expression and preservation of protein functions. When stimulated with IL-6 and IFN-γ, the cells showed fast and robust nuclear translocation of both RFP-STAT3 and STAT1-GFP with RFP-STAT3 being almost two times faster with a characteristic redistribution time of 7.6 minutes vs. 4.8 minutes for STAT1-GFP (Figure 4 Movie S1). The nuclear staining with 1 µM of a vital nuclear marker Hoechst 33342 did not interfere with STAT’s activation and nuclear translocation (Movie S1 Figure S1) and was used for FLIN measurements. The cytokine activation was very specific: IL-6 activated only RFP-STAT3 while IFN-γ activated only STAT1-GFP (Figure 5 Movie S2-S3). The pleiotropic cytokine IFN-β-1a activated both STATs (data not shown). Other tested cytokines and growths factors (IL-10, TGF-β1, TGF-α and EGF) were not effective for either STAT (data not shown, Table 1). The expression level of both tagged STATs is high enough to be detectable using a 20x high NA air objective that is common for high-throughput fluorescence microscopy (Figure S1).

**Table 1:** Comparison of the effect of various cytokines on activation of endogenous STAT1/STAT3 in the knock-in A549 cell line.

Ligand	Localization(C – cytoplasmic, N – nuclear)	
	STAT1-GFP	RFP-STAT3
Control	C	C/N
IL-6	C	N
IFN-γ	N	C/N
IL-10	C	C/N
IFN-β-1a	N	N
EGF	C	C/N
TGF-β1	C	C/N
TGF-α	C	C/N

### STAT3 inhibitor test

To test the sensitivity of the reporter cell line to compounds known to block STAT3 activation, the cells were pre-incubated with a STAT3 inhibitor for 1 hour at 37^°^C and then stimulated with the IL-6 /IFN-γ mixture (100 ng/ml each). Surprisingly, Stattic, which was discovered as a specific STAT3-SH2 domain inhibitor [37], completely stopped nuclear translocation of both RFP-STAT3 and STAT1-GFP (Figure S2). These findings are in agreement with other publications in which Stattic was also found to inhibit the phosphorylation of both STAT3 and STAT1 equally well [12,15]. Among several known STAT3 inhibitors tested in our model system (data not shown), we found Cpd3 that specifically interacts with the peptide-binding pocket of the STAT3 SH2 domain to be the most effective and least cytotoxic [36]. Starting at 10 µM this compound effectively suppressed IL-6-induced nuclear translocation of RFP-STAT3 while IFN-γ-induced redistribution of STAT1-GFP was not impaired (Figure 6B Movie S4).

### Cyclin D1 promoter activity test

Nuclear retention of activated STAT3 is transient as the activated transcription factor disappears from the nucleus within 1–6 h after ligand stimulation [38]. However, during its residence in the nucleus, STAT3 initiates expression of multiple downstream targets including genes involved in cell cycle progression (Cyclin D1, Fos, c-Myc, etc.) and anti-apoptotic genes (Survivin, Bcl-2, Bcl-xL and others) [39]. A potential STAT3-DNA binding inhibitor should selectively prevent this downstream gene activation. Ideally such an inhibitor should not interfere with STAT3 phosphorylation, dimerization and nuclear translocation but should block the downstream transcription activation and corresponding protein production. In order to screen for those inhibitors that specifically prevent a particular downstream target from being induced by activated STAT3, the target’s expression has to be reported in addition to monitoring the STAT3 activation. This can be accomplished if STAT3/STAT1 FP tagging is combined with luciferase reporting of the downstream target’s expression in the same cells.

To facilitate tracking of Cyclin D1 expression activated by STAT3-bound ISRE’s, we inserted a secreted luciferase reporter [35] (sLUC) into the CCND1 locus (Figure 1C). The luciferase insertion was done by using a ZFN in the RFP-STAT3/STAT1-GFP cell line. Triple knock-in clones were isolated and checked for correct donor integration (Figure 2). This system allows monitoring the activity of the three genes in the same cells. Cyclin D1 expression was measured by analyzing the aliquots of the cell medium. The addition of Cpd3 significantly down-regulates Cyclin D1 (Figure 7A) and inhibits cell growth (Figure 7B). The presence of Cpd3 (15-30 µM) completely impairs the cell division cycle after a few hours of incubation. In the long-term experiments (~20 hours) we observed multiple instances when cells entered the division cycle (i.e. detached from substrate and became spherical), but were “frozen” in metaphase after chromosomal alignment. Cells without Cpd3 grew normally (Movie S5).

## Discussion

Conventional transfection techniques allow expressing FP-tagged recombinant proteins of interest but in most cases this leads to overexpression of the fusion proteins. The overexpression itself might create strong artifacts in intracellular protein traffic and protein-mediated processes [5]. Moreover standard transfection methods are not ideal for FP-tagging of multiple proteins due to extremely high heterogeneity of exogenous protein expression within a population of cells. In contrast, using ZFN technology allows simultaneous detection of multiple FP-tagged targets, making this a promising approach for drug discovery. Tagging multiple endogenous proteins of interest with FPs of different colors will help to overcome overexpression artifacts because the fusion proteins will be expressed at native levels and will be controlled by complex endogenous regulation.

We used ZFN technology to generate a triple reporter A549 cell line (RFP-STAT3/STAT1-GFP/sLUC-2A-CCND1 cell line) by inserting three different reporters into three different loci (Figures 1-2) to follow the activities of these three targets. Native gene expression and regulation could be monitored in this triple knock-in cell line in contrast to cell lines with target overexpression under an exogenous promoter as in the case of transient transfection or stable cell lines with random integration.

To assess the expression and phosphorylation of FP fusion proteins (~120 kDa) relative to the wild type (~90 kDa) for STAT1 and STAT3, we performed Western analysis of the generated cell line with and without stimulation by IL-6 and IFN-γ (Figure 3). The aneuploid A549 cells have a very unstable genome with copy number for both STAT1 and STAT3 genes being at least three [40]. The data showed that at least one copy was tagged and at least one copy was not modified in both cases. These results were confirmed by PCR with genomic primers flanking the integration site (data not shown).

In the case of STAT1 after IFN-γ treatment there was a strong band on our Western blots corresponding to phosphorylated untagged STAT1. Further analysis indicated the presence of a double band (resolved in Figure 6) showing expression and phosphorylation of both STAT1 isoforms (91 and 84 kDa [6]). C-terminal insertion of GFP tags only the α isoform of STAT1 and most probably only one copy of it. Therefore the phosphorylated STAT1-GFP migrated as a single species that was weaker than the untagged version (Figures 3, 6). In the case of STAT3 where both isoforms (92 and 89 kDa [6]) were tagged, the RFP-STAT3 migrated as two distinct proteins (resolved for total STAT3 in Figure 6).

Surprisingly, the untagged STAT3 was expressed but was not phosphorylated after IL-6 application in the knock-in cells as only RFP-STAT3 was detected by Western analyses with a phospho-STAT3 antibody. However, total STAT3 consisted of both tagged and untagged isoforms (Figures 3, 6), which could be explained if we assume that in engineered cells the majority of unphosphorylated STAT3 exists as STAT3: RFP-STAT3 heterodimers. Since unphosphorylated STATs dimerize in the antiparallel conformation [6], the RFP tag at the N-terminus of the tagged monomer will be positioned very close to the C-terminus of the untagged monomer in the heterodimer. Then RFP could sterically protect the SH2 domain and the tyrosyl tail segment of the untagged monomer preventing it from being phosphorylated while the C-terminus of the tagged monomer remains accessible to Jak kinases.

When stimulated with IL-6 or IFN-γ, the cells showed fast and robust nuclear translocation of RFP-STAT3 or STAT1-GFP, respectively. If stimulated with the IL-6/IFN-γ mixture, both STATs redistribute into the nucleus: first STAT3 then STAT1 (Figure 4 Movie S1). The wild type non-small-cell lung tumor A549 cells harbor aberrantly active STAT3 [41–43]. Indeed we observed that before the stimulation with IL-6 a significant number of cells in the knock-in line population already have some RFP-STAT3 localized in the nucleus. This baseline red nuclear staining becomes more pronounced upon stimulation. There is no cross-talk between IL-6 and IFN-γ (STAT3 and STAT1) channels meaning that IL-6 can activate only STAT3 and IFN-γ – only STAT1 (Figures 3, 5 Movie S2-S3).

Effective and selective STAT3 inhibitors could be distinguished by observing only GFP in nuclei, as they should impair only RFP-STAT3 nuclear translocation without effecting STAT1-GFP (the case of Cpd3: Figure 6 Movie S4). However, if a tested compound stops STAT1 redistribution instead of STAT3, the nuclei will become red. With compounds that inhibit both STATs, the nuclei will stay unchanged after stimulation. All experiments where green nuclei are detected should be considered as strong leads, i.e. the selective STAT3 inhibitors. Since the cells are still alive after the experiment (no fixation is required), a washout step followed by a second stimulation could provide information about the reversibility of the inhibitor.

Inhibitors that prevent Cyclin D1 from being induced by activated STAT3 could be revealed by the luminescence readings. In the case of Cpd3, Cyclin D1 got significantly down-regulated (Figure 7A) and cell growth was inhibited (Figure 7B). It was reported that the suppression of Cyclin D1 with antisense induces both cell growth arrest and apoptosis [44,45]. Also, it was shown that the knockdown of STAT3 expression by RNA interference (RNAi) suppresses the growth of SKOV3 cells and arrests the cell cycle in the G1 phase [46]. Consistent with our findings, the authors mentioned that “cells transfected with siRNA-stat3 became less confluent and some cells were rounded and detached from the plates, when compared with cells transfected with scrambled vectors”. Our monitoring of the effect of Cpd3 on the cell cycle by time-lapse imaging revealed aberrations in cell division (Movie S5).

The A549 RFP-STAT3/STAT1-GFP/sLUC-2A-CCND1 cell line provides a simple and convenient method for multiplexed high-content screening and pre-clinical testing of potential STAT3 inhibitors while ensuring that the STAT1 pathway is not affected. All drug leads should be revealed during a one step treatment: 1 hour pre-incubation with the inhibitor followed by the stimulation of the cells with the IL-6/IFN-γ mixture. In addition to the fast and robust response, this model system has other important advantages: 1) all cell-impermeable compounds will be eliminated; 2) the cytotoxic compounds will be revealed; 3) IC_50_ for lead compounds can be measured *in situ*; 4) the effect on downstream STAT3 targets (Cyclin D1) can be determined (Figure 7); 5) wells with the lead compounds where the cells have green nuclei might be monitored for hours or days for signs of possible apoptosis or could be tested using any apoptotic assays or markers.

Our cell line might also be valuable for finding compounds other than just those that inhibit STAT3 in cancer. Changes in STAT3 activity have been found in a variety of other human diseases [12]. Thus STAT3 modulators could be discovered and tested in models for those disorders. For example zoniporide was shown to be cardioprotective through activation of STAT3 in a rat myocardial preservation model [47]. Our cell line might also be valuable for finding compounds that selectively activate the STAT1 pathway. Activated STAT1 induces anti-proliferative and pro-apoptotic genes that directly hamper tumor growth [48].

The combination of STAT3/STAT1 FP-tagging with reporting of one STAT3 downstream target gene activity by secreted luciferase will allow screening for many kinds of possible STAT3 inhibitors, including the selective DNA-binding inhibitors that block ISRE. Thus this approach of ZFN–tagging multiple gene targets in a single cell line allows for the creation of effective cell-based assays for compound screening.

## Supporting Information

Figure S1 A vital nuclear marker Hoechst 33342 did not interfere with endogenous STAT1/STAT3 nuclear translocation upon simultaneous activation. Fluorescence microscopy images of an isolated single cell clone expressing the endogenous STAT1 protein tagged with GFP at the C-terminus and the endogenous STAT3 protein tagged with RFP at the N-terminus (A549 lung carcinoma). The cells were imaged live after addition of 100 ng/mL IFN-γ and 100 ng/mL IL-6 using a 20x/0.75 air objective. The cells were preincubated with 1 µM of Hoechst 33342. The scale bar is equal to 50 µm.
(TIF)

Figure S2 Stattic inhibits endogenous activation of both STAT1 and STAT3. Fluorescence microscopy images of an isolated single cell clone expressing the endogenous STAT1 protein tagged with GFP at the C-terminus and the endogenous STAT3 protein tagged with RFP at the N-terminus (A549 lung carcinoma). Cells were pre-incubated for 1 hour with 20 µM Stattic, a specific STAT3 inhibitor. The addition of a mixture of 100 ng/ml each of IL-6 and IFN-γ did not induce STAT1 nuclear translocation. Some residual STAT3 translocation could be seen. The STAT3 and STAT1 images were taken 40 minutes after addition of the receptor ligands. The cells were imaged live using a 40x/1.3 oil objective. The scale bar is equal to 25 µm. The Cpd3 structure is shown in the upper left corner.
(TIF)

Movie S1 Endogenous STAT1/STAT3 nuclear translocation upon simultaneous activation (see legend for Figure 4). The cells were preincubated with 1 µM of Hoechst 33342.
(AVI)

Movie S2 Upstream ligand selectivity for activation of endogenous STAT1/STAT3: 100 ng/mL of IL-6 was added 29 minutes after IFN-γ (see legend for Figure 5A). (AVI)

Movie S3 Upstream ligand selectivity for activation of endogenous STAT1/STAT3: 100 ng/mL of IFN-γ was added 30 minutes after IL-6 (see legend for Figure 5B). (AVI)

Movie S4 Cpd3 selectively inhibits activation of endogenous STAT3, not STAT1. Cells were pre-incubated for 1 hour with 10 µM Cpd3, a specific STAT3 inhibitor (See legend for Figure 6B).
(AVI)

Movie S5 Cpd3 effect on the reproduction cycle of wild type A549 cells monitored by time-lapse imaging. Differential Interference Contrast (DIC) images were acquired every 5 minutes for 19 hours using a 20x/0.75 air objective. Cpd3 treated cells were pre-incubated with 30 µM Cpd3 for 1 hour before starting the acquisition.
(AVI)
