# Correction: Tagging of Genomic STAT3 and STAT1 with Fluorescent Proteins and Insertion of a Luciferase Reporter in the Cyclin D1 Gene Provides a Modified A549 Cell Line to Screen for Selective STAT3 Inhibitors

**DOI:** 10.1371/annotation/904d2af7-5732-402d-8d85-15128c83ea0f

**Published:** 2014-01-22

**Authors:** Andrey Samsonov, Nathan Zenser, Fan Zhang, Hongyi Zhang, John Fetter, Dmitry Malkov

The legend for sub-figure 2B was incorrectly placed directly below the text box for Figure 2.

