# Supplementary figures and images for: Tagging of Genomic STAT3 and STAT1 with Fluorescent Proteins and Insertion of a Luciferase Reporter in the Cyclin D1 Gene Provides a Modified A549 Cell Line to Screen for Selective STAT3 Inhibitors
Source: PLoS One. 2013 Jul 9;8(7):e68391. doi: 10.1371/journal.pone.0068391 (PMC3732202; doi:10.1371/journal.pone.0068391)

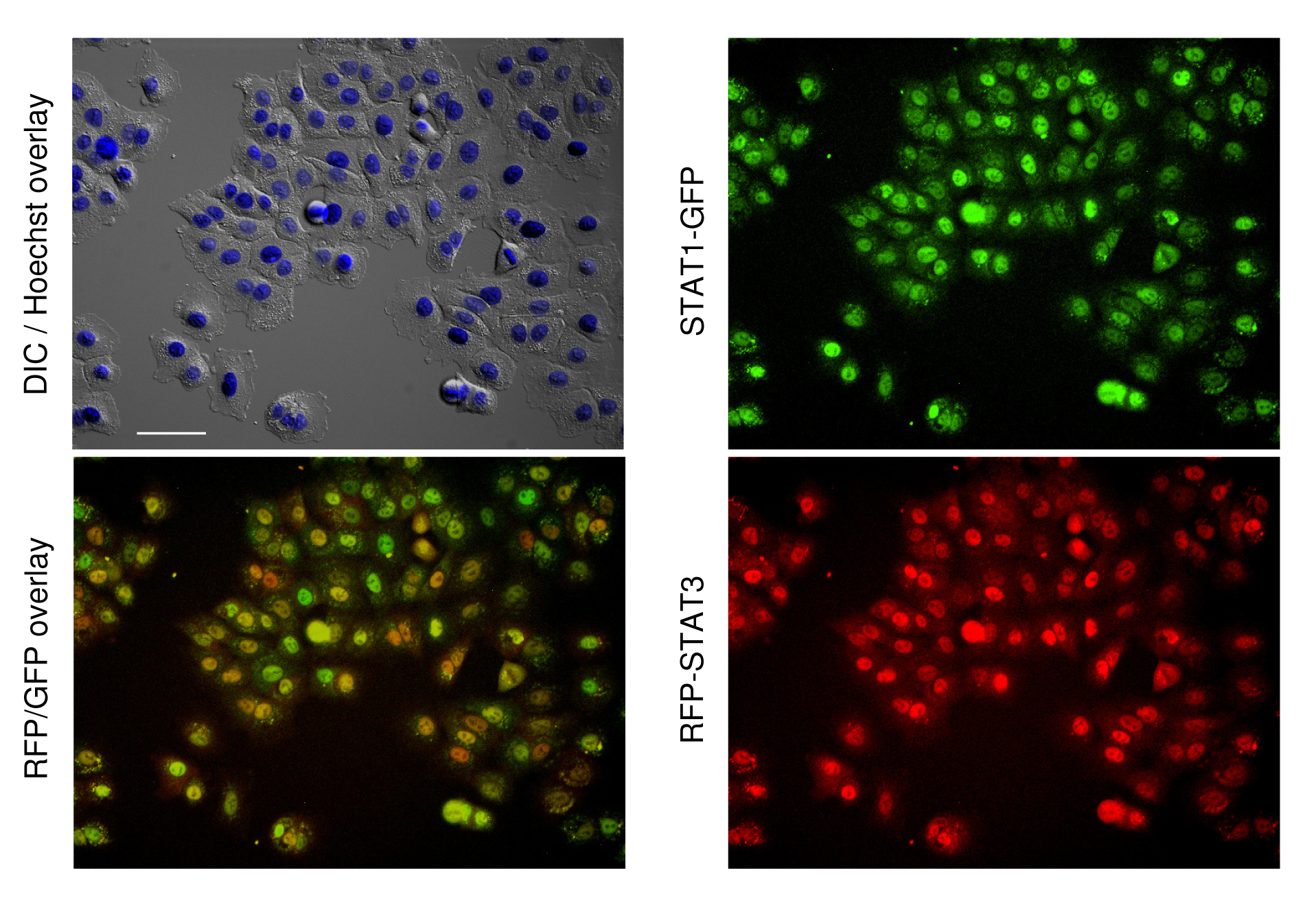

Supplement: Figure S1 — Fluorescence microscopy images of an isolated single cell clone expressing the endogenous STAT1 protein tagged with GFP at the C-terminus and the endogenous STAT3 protein tagged with RFP at the N-terminus (A549 lung carcinoma). The cells were imaged live after addition of 100 ng/mL IFN-γ and 100 ng/mL IL-6 using a 20x/0.75 air objective. The cells were preincubated with 1 µM of Hoechst 33342. The scale bar is equal to 50 µm. (TIF) [file pone.0068391.s001.tif]

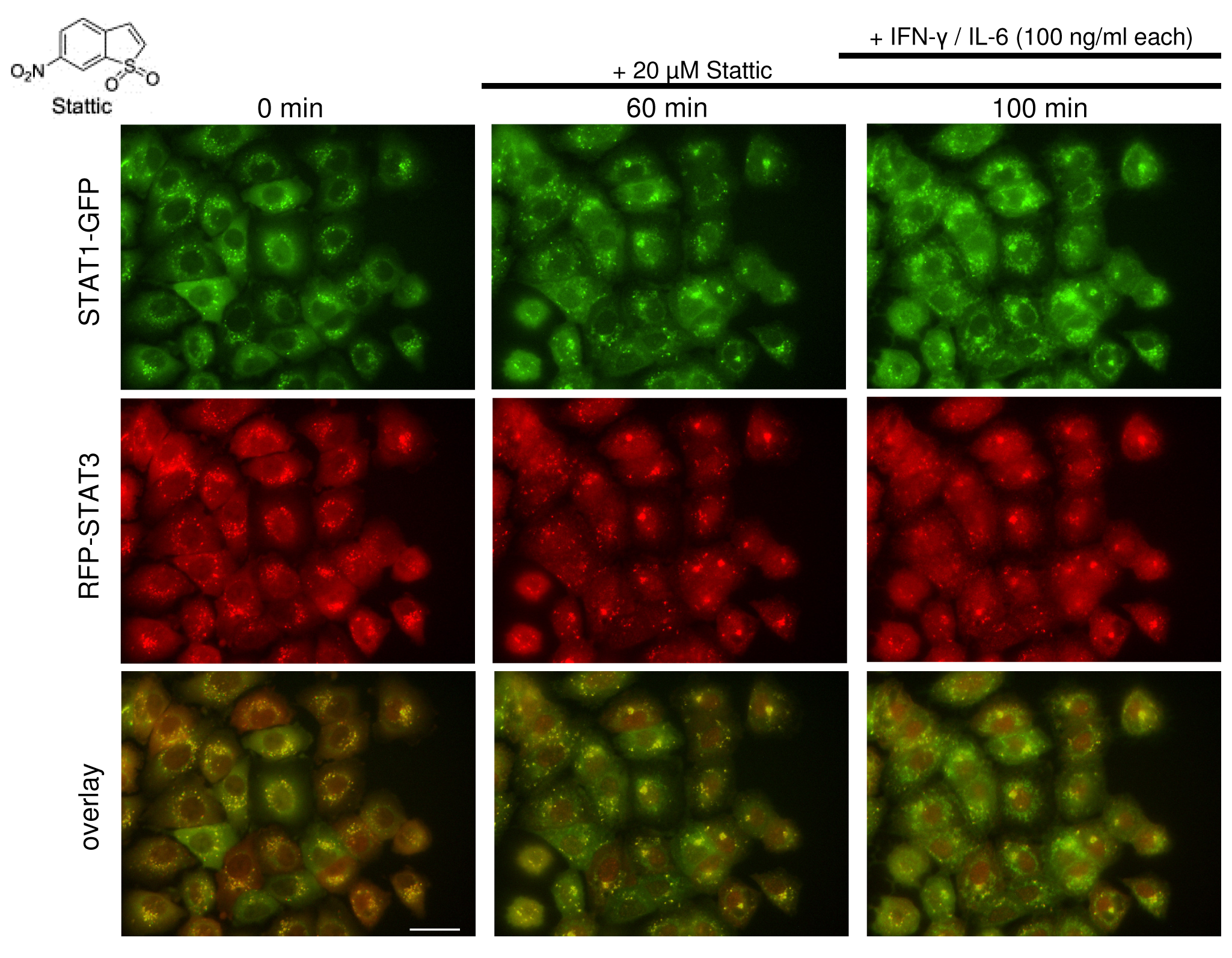

Supplement: Figure S2 — Fluorescence microscopy images of an isolated single cell clone expressing the endogenous STAT1 protein tagged with GFP at the C-terminus and the endogenous STAT3 protein tagged with RFP at the N-terminus (A549 lung carcinoma). Cells were pre-incubated for 1 hour with 20 µM Stattic, a specific STAT3 inhibitor. The addition of a mixture of 100 ng/ml each of IL-6 and IFN-γ did not induce STAT1 nuclear translocation. Some residual STAT3 translocation could be seen. The STAT3 and STAT1 images were taken 40 minutes after addition of the receptor ligands. The cells were imaged live using a 40x/1.3 oil objective. The scale bar is equal to 25 µm. The Cpd3 structure is shown in the upper left corner. (TIF) [file pone.0068391.s002.tif]
